# Myelin Fat Facts: An Overview of Lipids and Fatty Acid Metabolism

**DOI:** 10.3390/cells9040812

**Published:** 2020-03-27

**Authors:** Yannick Poitelon, Ashley M. Kopec, Sophie Belin

**Affiliations:** Department of Neuroscience and Experimental Therapeutics, Albany Medical College, Albany, NY 12208, USA; poitely@amc.edu (Y.P.); kopeca@amc.edu (A.M.K.)

**Keywords:** Schwann cell, oligodendrocyte, myelin, lipid, fatty acid

## Abstract

Myelin is critical for the proper function of the nervous system and one of the most complex cell–cell interactions of the body. Myelination allows for the rapid conduction of action potentials along axonal fibers and provides physical and trophic support to neurons. Myelin contains a high content of lipids, and the formation of the myelin sheath requires high levels of fatty acid and lipid synthesis, together with uptake of extracellular fatty acids. Recent studies have further advanced our understanding of the metabolism and functions of myelin fatty acids and lipids. In this review, we present an overview of the basic biology of myelin lipids and recent insights on the regulation of fatty acid metabolism and functions in myelinating cells. In addition, this review may serve to provide a foundation for future research characterizing the role of fatty acids and lipids in myelin biology and metabolic disorders affecting the central and peripheral nervous system.

## 1. Introduction

**Figure 1:**
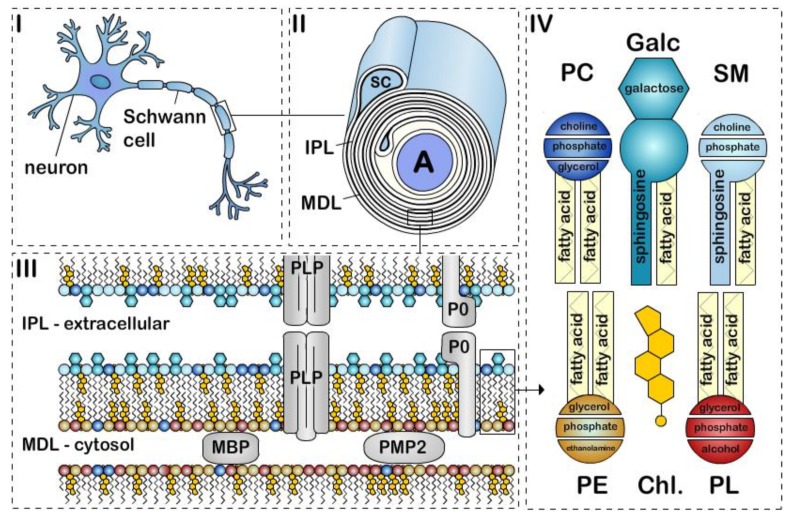
The structure of peripheral nervous system myelin sheath. Schematic representation of a (**i**) myelinated axon, (**ii**) myelin sheath, (**iii**) bilayer membrane, and (**iv**) major lipids classes. Myelin is formed by apposition of the external surfaces and internal surfaces of the myelin bilayer that constitute the intraperiodic line (IPL) and the major dense line (MDL), respectively (**ii**, **iii**). The myelin bilayer has an asymmetric lipid composition (**iii**, **iv**). Myelin protein are also asymmetrically distributed with for example PLP and P0 in the IPL and MBP and PMP2 at the MDL (**iii**). Cholesterol (Chl.), Galactosylceramide (Galc, cyan), Plasmalogen (PE, yellow), Phosphatidylcholine (PC, dark blue), Sphingomyelin (SM, light blue) and other phospholipids (PL, red). P0, PMP2 proteins and the enrichment of sphingomyelin in the myelin are specific to PNS myelin.

Myelin is a specialized multilamellar membrane consisting of 40 or more tightly wrapped lipid bilayers [1]. The deposition of compact myelin in a spiraling pattern around an axon generates two morphological features that can be observed by electron microscopy, (1) the major dense line (MDL), which is the tight apposition of the cytoplasmic leaflets, and (2) the intraperiod line (IPL), which is the apposition of the extracellular leaflets (Figure 1). Myelin is made by oligodendrocytes in the central nervous system (CNS) and Schwann cells in the peripheral nervous system (PNS). CNS and PNS myelin differ in several important ways. One oligodendrocyte forms myelin sheath segments for several neurons, whereas a single Schwann cell myelinates one segment for a single neuron. During the development of the vertebrate nervous system, myelination starts around birth first in the PNS, then the spinal cord and finally in the brain. Although most myelination will be completed in the PNS shortly after birth (within two years after birth in humans; within four weeks after birth in rodents), myelination in the CNS is an ongoing process that continues throughout adulthood. During active CNS myelination in rodents, the myelin sheath expands at a rate 5 × 10^3^ – 5 × 10^4^ μm^2^/cell/day (15 to 150 times faster than a normal cell body membrane extension). In addition, one oligodendrocyte or one Schwann cell supports a surface area of myelin up to 2 mm^2^ or 20 mm^2^ respectively, which can represent 2000 times the cell surface area of an epithelial cell [2,3]. Although the turnover of myelin lipids in humans is uncharacterized, studies in mice showed that lipids within the myelin sheath are continuously remodeled, and lipid turnover rates are differently regulated through life [4]. Thus, biosynthesis, storage, and cellular trafficking of myelin lipids are essential to the assembly and maintenance of myelin in the nervous system through life span. Recent reviews have provided updates on the genes, proteins, and molecular signals controlling myelination both in the PNS and CNS [5,6,7]. Herein, we focus on the most recent developments regarding myelin lipids and fatty acids.

## 2. Myelin Lipids—Role of Lipids in Myelin

The myelin sheath is characterized by a high proportion of lipids (70%–85%) and consequently a low proportion of proteins (15%–30%). In contrast, most biological membranes have approximatively equivalent ratio of proteins to lipids (50% lipid/50% protein) [8]. The high lipid/protein ratio in myelin contributes to the close packing and tight organization of the myelin sheath through non-covalent interactions between lipids and myelin proteins [9]. In addition, the enrichment in specific classes of lipids is also required for the long-term maintenance of myelin [10]. The three major classes of membrane lipids are cholesterol, phospholipids (e.g., plasmalogen, lecithin, sphingomyelin) and glycolipids (e.g., galactosylceramide). The lipid composition of myelin sheath is distinctive, made of high amounts of cholesterol and enriched in glycolipid, in a ratio of 40%:40%:20% (cholesterol, phospholipid, and glycolipid, respectively) compared to most biological membranes (25%:65%:10%) [11]. 

Myelin is not a simple homogeneous layer of proteins and lipids. It also contains discrete and dynamic lipid domains in the external leaflet of its plasma membrane called lipid rafts [12,13]. Lipid rafts are characterized by the concentration of selected membrane lipids such as cholesterols, galactosylceramide, and low levels of phosphatidylcholine. These lipids have roles in myelin formation and stabilization [14,15] by including and excluding specific proteins into particular compartments and orchestrating membrane protein trafficking [16,17] and subsequent signal transduction [18] (see [16,19] for review). In CNS and PNS myelin, lipid rafts have been suggested to promote cellular adhesion by facilitating the localization of membrane and transmembrane proteins mediating axon-glia recognition [14,20,21,22,23,24].

While there are no lipids that are specific to the myelin [25], the three most abundant lipids are (i) cholesterol, (ii) galactosylceramide, and (iii) plasmalogen. Together, these three lipids comprise 65% of the total myelin lipids. Lipid composition between CNS and PNS myelin are remarkably similar, with the exception of (iv) phosphatidylcholines and (v) sphingomyelin levels which are relatively abundant in the peripheral myelin sheath compared to the central myelin sheath [25,26] (Table 1). 

## 3. Cholesterol

The brain contains about 20% of the body’s cholesterol, which makes it the richest cholesterol-containing organ [29]. The largest pool of free cholesterol in mammals resides in the myelin [30] (Table 1). In myelin, cholesterol inserts into the membrane bilayers to increase myelin viscosity and stabilize myelin lipids and proteins [31]. Myelin cannot be synthesized without cholesterol, and cholesterol availability is a critical prerequisite and a limiting factor of myelin membrane growth during CNS maturation [32]. Due to the blood–brain barrier (BBB) in the CNS, the cholesterol present in myelin mostly comes from de novo synthesis in oligodendrocytes or neighboring astrocytes [32,33,34,35]. The rate of cholesterol synthesis is highest during periods of active myelination, and following completion of myelination the production of cholesterol drops by 90% [36,37]. The incorporation of cholesterol into myelin membranes starts, like most lipids, in the endoplasmic reticulum. Cholesterol molecules tightly assemble into lipid rafts, combine with integral myelin proteins in the Golgi and reaches the myelin through vesicular [15,38,39], and possibly non-vesicular transport mechanisms [40]. Once cholesterol is integrated into myelin, the renewal of cholesterol is much slower, with a half-life around 5 years in adult human brain [41].

Several studies have further highlighted the importance of cholesterol synthesis and transport in myelinating cells. Myelin synthesis and oligodendrocyte differentiation is severely perturbs in oligodendrocytes unable to synthesize cholesterol, despite the demonstrated ability of oligodendrocytes to uptake cholesterol from neighboring cells, such as astrocytes [32,42]. Moreover, knockout animals models with deletion of the Niemann–Pick disease type C protein (NPC1), a protein mainly involved in the intracellular transport of cholesterol, exhibit progressive demyelination of the CNS similar to the human condition [43,44]. In the PNS, Schwann cells do not suffer from the same restrictions imparted by the BBB on oligodendrocytes, and can uptake cholesterol from circulation [45]. However, Schwann cells also rely primarily on de novo cholesterol synthesis [46]: Schwann cells unable to synthesize cholesterol fail to myelinate or produce only thin myelin sheaths [47,48]. Interestingly, a recent study showed that a transcription factor (Maf) downstream of Neuregulin signaling regulates cholesterol synthesis in Schwann cells, suggesting that extrinsic axonal signals are regulating Schwann cell cholesterol biosynthesis [49]. Cholesterol is also a precursor for oxysterols (cholesterol oxide derivatives), often found in abnormal levels in patients with neurodegenerative diseases. While oxysterols can accumulate in plasma membrane, their role in regulating myelin production is only speculative [50].

## 4. Galactosylceramide

Galactosylceramide and its sulfated form, sulfatide, are two glycosphingolipids that are highly and uniquely enriched in both oligodendrocytes and Schwann cells. Interestingly, galactosylceramide is more abundant in compact myelin while sulfatide is mainly located in noncompact myelin [51]. Together they account for about 20% of the total myelin lipids in oligodendrocytes (Table 1) [32,52,53]. Galactosylceramides present in the myelin bilayer preferentially consisting of (1) a galactose constituting the head group, (2) a sphingosine-based backbone, and (3) a very long-chain fatty acids tail group [54,55,56] (Figure 1). Galactosylceramides are extremely hydrophobic molecules, and among myelin lipids, they contribute the most to myelin formation and stability. Together with highly hydrophobic myelin proteins, they generate important hydrophobic forces between the myelin membranes. These intermolecular hydrophobic forces contribute to myelin membrane “zippering”, by creating attractive forces that bring myelin membranes into close contact, and repulsive force toward extracellular and cytosolic fluids [57,58]. In particular, opposing galactose heads of galactosylceramides at the IPL have additional attractive properties (Figure 1) [59,60]. Galactosylceramides also have a high proportion of long-chain fatty acids that intercalate into the inner membrane leaflet [58], which serve to further increase myelin membrane stability. Synthesis of galactosylceramide in myelinating cells takes place in the endoplasmic reticulum, once integrated into the myelin, their half-life varies from 3–8 months in the mouse brain [61].

While galactosylceramides are important for myelin formation, they are not essential, and their absence can be partially compensated by production of other glycolipids such as glucosylceramide [58,62]. In animals unable to synthesize galactosylceramide, myelination is relatively normal except for thinner myelin and occasional myelin splitting [63,64]. Similarly, mice lacking the major form of galactosylceramides in myelin (2-hydroxlyated galactosylceramide and sulfatide) myelinate normally and only developed signs of myelin degeneration by 18 months of age, and mice lacking galactosylceramide sulfatide showed minor disorganization of uncompact myelin regions [65,66,67,68,69].

## 5. Plasmalogen 

Plasmalogens are a subclass of phospholipids, mainly found in the cell membranes, and categorized by either a choline or ethanolamine head group. Ethanolamine plasmalogens are the predominant phospholipids found in myelin (Table 1). They are composed by (1) an ethanolamine head group, (2) a glycerophosphoric acid backbone, and (3) fatty acids tails (Figure 1). Although the functions of plasmalogens have not yet been fully elucidated, they are proposed to contribute to strengthening bonds with adjacent lipids and enable a more compact and stable myelin [58,70]. In addition, a recent study suggested that plasmalogens may be crucial to protect myelin against the oxidative stresses associated with aging [71]. Plasmalogen biosynthesis is initiated in the peroxisome and completed in the endoplasmic reticulum, after which they are transported asymmetrically to the inner leaflet of the myelin membrane [72]. In humans, plasmalogen levels in the myelin increase until about 30–40 years of age and then dramatically decline around 70 years of age [73]. In addition, the half-life of plasmalogens in adult rat myelin is much shorter (10–30 days) than other myelin lipids [4,74].

In the CNS, in mice deficient for key enzymes regulating plasmalogen biosynthesis (i.e., *P*ex*7*, *A*bcd*1*, or *G*npat), myelination proceeds normally, but a reduced amount of myelin and disorganized paranodes has been observed [75,76,77]. In contrast, in the PNS, plasmalogens are important for two fundamental events of Schwann cell development: axon-glia recognition and myelination. Plasmalogen-deficient Schwann cells present an impairment of AKT activation at the plasma membrane [78] and AKT signaling is known to be necessary for proper Schwann cell development during axon–glia recognition and myelination [1,79].

## 6. Phosphatidylcholine

Phosphatidylcholines (also called lecithin) are an abundant phospholipid found in myelin, particularly in the PNS (Table 1). Phosphatidylcholines are composed by (1) a choline head group, (2) a glycerophosphoric acid backbone, and (3) fatty acids tails (Figure 1). They are structural components of the myelin, with functions in initiation, compaction and maintenance of plasma membrane [80]. In oligodendrocytes and Schwann cells, the predominant pathway to synthesize phosphatidylcholines relies on de novo synthesis through choline uptake [81]. Phosphatidylcholines preferentially integrate in the outer layer of the myelin sheath; however, previous studies in Schwann cells have shown that phospholipids that are integrated first in outer layers of the myelin may move within the membrane to the inner layer [82,83]. Similarly to plasmalogens, phosphatidylcholines have a very short half-life on the order of days to weeks [4,84].

While the effects of deficiency in phosphatidylcholine synthesis on myelination has not been studied thus far, recent studies indicate that choline homeostasis may be associated with myelination during development and myelin repair [81,85]. Also, phosphatidylcholines are precursors for the synthesis of other important classes of signaling and structural phospholipids, the sphingomyelin, which share the same head group, and the phosphatidylinositols and their phosphorylated forms, which all are critical to PNS myelination [86,87,88,89,90,91,92]. 

## 7. Sphingomyelin

Sphingomyelin are a lipid class enriched in PNS myelin. They consist of phosphocholine head group associated to a sphingosine-based backbone, comparable to the lipid backbone found in galactosylceramide (Figure 1) [93]. Their functions are also very similar to galactosylceramide, as they promote membrane interactions within the myelin structure [94,95] In addition to its structural role in the myelin, sphingomyelin is also involved in signal transduction pathways [96,97] and the regulation of cholesterol and protein trafficking to the myelin [15,85,98,99]. In most cells sphingomyelin synthesis occurs primarily in endoplasmic reticulum and Golgi, but in oligodendrocytes, about 50% sphingomyelin is synthesized at the plasma membrane, indicating a cell type-specific subcellular localization for sphingomyelin formation [96,100]. However, the precise topology and physiological roles of sphingomyelin synthesis in myelinating cells remain virtually unstudied. Sphingomyelin integrates primarily in the outer leaflet of the plasma membrane and, in the rat CNS, they have an extended half-life over 15 months [101].

Mice deficient for sphingomyelin synthesis (*S*ms*1* or *S*ms*2* constitutive knockout) were reported to be normal, with no myelin defect [102,103]. In contrast, deletion or inhibition for the enzyme responsible for sphingomyelin hydrolysis into phosphatidylcholine and ceramide (sphingosine linked to a fatty acid), causes a significant increase in myelin recovery in animals treated with cuprizone, indicating that sphingomyelin may have beneficial functions in the myelin sheath repair [104].

## 8. Fatty Acid Metabolism in Myelinating Cells

Myelin lipids, apart from cholesterol, all use fatty acids as part of their fundamental structure (Figure 1). Because myelin requires a high amount of fatty acids for its assembly and maintenance, myelinating cells are particularly vulnerable to fatty acid and lipid disorders (see [10,95] for reviews on human myelin disorders associated with fatty acid or lipid synthesis). Fatty acids can differ by length (from 2 to 30 carbons) and by the chemistry (degree of saturation) of their hydrocarbon chain, both of which can alter the fluidity of myelin membrane [105]. Myelin contains high levels of saturated very long chain fatty acids (VLCFA) [106]. The intermolecular interactions between long fatty acid tails add rigidity to the membrane. Saturated fatty acid tails have no double bonds and as a result are straight, which maximizes the interactions between lipids saturated fatty acid tails. Thus, a high content of saturated VLCFA functions to decrease myelin fluidity and provide a thick permeability barrier for ions to insulate axon [95].

Most fatty acids can be synthesized autonomously by the cell and are thus considered to be non-essential fatty acids. Fatty acids that need to be provided by diet are referred to as essential fatty acids. In addition to their structural function in lipids, fatty acids are important substrates for energy generation and present an important alternative to glucose. Fatty acids are also used for the synthesis of neuromodulatory lipids, e.g., prostaglandins. Critical pathways of fatty acid regulation in myelinating cells are currently being explored, including (i) fatty acids synthesis (for the synthesis of myelin lipids), (ii) fatty acids uptake, and (iii) fatty acid oxidation (as energetic source for oligodendrocytes, Schwann cells and axons). 

## 9. Fatty Acid Synthesis

**Figure 2:**
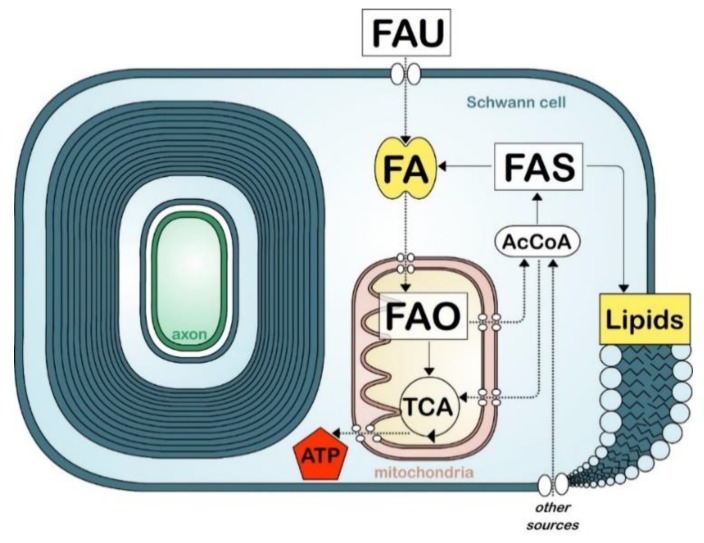
Simplified view of fatty acid metabolism in myelinating cells. Fatty acid (FA) uptake (FAU), synthesis (FAS), and oxidation (FAO). Tricarboxylic acid cycle (TCA). Acetyl-coA (AcCoA). Other sources include glucose and amino acids.

Because of their high rates of membrane production during myelination, the high lipid content and specific lipid composition of their membranes, oligodendrocytes and Schwann cells rely heavily on fatty acid synthesis. This process is generally initiated with the carboxylation of acetyl-CoA, derived from carbohydrates via the glycolytic pathway, into malonyl-CoA (Figure 2). Acetyl-CoA and malonyl-CoA are then used by fatty acid synthase, an enzyme system that catalyzes the de novo synthesis of medium and long chain fatty acids (up to 16 carbons). The expression of fatty acid synthase correlates with myelination during development and is regulated by sterol regulatory element-binding proteins 1 (SREBP1) and SREBP cleavage activating protein (SCAP) [48,107]. SREBP1, like other SREBPs, is activated by a reduction of intracellular cholesterol, indicating a homeostatic link between fatty acid synthesis and cholesterol synthesis [108,109]. De novo fatty acid synthesis is critical for the correct formation and growth of myelin both in the PNS and in the CNS [33,48,110,111,112]. Animals ablated for fatty acid synthase (encode by *F*asn) in either Schwann cells or oligodendrocytes present a partial block in the onset and efficiency of myelination. However it is unclear if the myelination defects observed in these animals are solely caused by an impairment of lipid synthesis, or by the impairment of other lipid-mediated functions such as fatty acid oxidation or transcriptional regulation [113]. Limitations in fatty acid synthesis have also been studied indirectly by examining the synthesis of fatty acids precursor, acetyl-CoA. For instance, (1) myelin defects are observed in the PNS of animals with disrupted Schwann cell mitochondria [114,115]. Authors suggested that these defects were caused in part by a switch from fatty acid synthesis to fatty acid oxidation. Also (2), mice deficient for a lactate transporter in Schwann cells showed thinning of myelin in sensory fibers associated with a reduced fatty acid and sphingosine synthesis [116]. In addition (3), myelin defects occurred with pyruvate dehydrogenase deficiency, an enzyme required for the synthesis of acetyl-coA from glycolytic sources [117,118]. However, a recent study reported that myelination is normal in animals with ablated pyruvate dehydrogenase in myelinating cells, indicating that oligodendrocytes and Schwann cells metabolism do not rely essential on glycolytic sources for the generation of acetyl-CoA [119]. 

The elongation of long chain fatty acids (over 16 carbons) into VLCFA (over 20 carbons) occurs in the endoplasmic reticulum. Compared to lipids from other plasma membranes, myelin lipids contain a high percentage of VLCFAs [95,120]. VLCFAs play a role in myelin maintenance and it is suggested that oligodendrocytes and Schwann cells have an ideal “set-point” for the amount of VLCFAs. Mice deficient for the synthesis of VLCFA present myelin defects [55]. Similarly, the abnormal accumulation of VLCFAs can also cause demyelination, either directly by having disruptive effects on the stability and structure of the myelin and/or indirectly by limiting the synthesis of plasmalogens in the peroxisome [77,121,122]. Also, mice lacking SREBP cleavage activation protein (SCAP) have reduced saturated VLCFA levels [48]. It was suggested that reduced saturation of VLCFAs contributes to myelin abnormalities observed in SCAP-null mice [95].

## 10. Fatty Acid Uptake

Besides fatty acid synthesis, myelinating cells have the ability to uptake fatty acids (Figure 2). In contrast to cholesterol or lipids, which are a larger class of molecules, fatty acids can be transported through the blood circulation, pass the BBB, and be transported through endothelial cells and astrocytes to myelinating cells (Figure 2) [123]. Fatty acids can passively diffuse through the plasma membrane or be actively recruited by fatty acid translocase (CD36) or fatty acid transport proteins (FATP) [124]. In addition, fatty acid binding proteins (FABPs), which are molecular chaperones for fatty acids, also enhance fatty acid uptake and trafficking to specific compartments in the cell (e.g., endoplasmic reticulum for membrane synthesis, mitochondria for oxidation) [125,126,127]. The role of CD36, FATPs and FABPs in oligodendrocyte lineage and CNS myelination is not well studied. While knockout models for these proteins exist, no studies have investigated their effect on myelin formation. Of note, while FATP1 is the predominant isoform of FATP expressed in the brain, oligodendrocytes and their precursors preferentially express FATP4 [128,129]. Oligodendocytes also express both FABP7 and FABP5 at different stages during their maturation, in oligodendrocyte precursor cells and in mature oligodendrocytes, respectively. In mice, ablation of *F*abp*7* lowers proliferation in oligodendrocytes and reduces their differentiation in immature oligodendrocytes. In contrast, ablation of *F*abp*5* did not affect oligodendrocytes proliferation or differentiation to immature oligodendrocytes, but decreases oligodendrocytes differentiation to mature myelinating oligodendrocytes [130]. Despite these changes, the authors did not report defects in myelin formation after the ablation of either of these FABPs.

In the PNS, there have been more studies on fatty acid uptake in Schwann cells. Ablation of *C*d*36* delays remyelination after nerve crush injury [131]. FABP8 (PMP2/peripheral myelin protein 2), along with P0 glycoprotein and myelin basic protein, is one of the major proteins in the peripheral nervous system myelin (up to 15% of myelin protein). PMP2 localizes in compact myelin at the MDL (Figure 1), and although PMP2 is not expressed by all Schwann cells, higher levels of PMP2 expression are observed in Schwann cells myelinating large diameter axons [132,133]. Despite its high expression levels in myelin, the physiological role of PMP2 remains unclear. PMP2 is unique in the FABP family, as it has stable contact to membranes and can stack lipid bilayers into highly ordered multilayers [134,135]. Thus, PMP2 was thought to function in myelin assembly, stabilization or turnover. However, animals ablated for *P*mp*2* do not develop major myelin alterations during development, in adulthood or after injury [133,136]. PMP2 has have high binding affinity to fatty acids and cholesterol [137,138] and is proposed to participate in fatty acid transport and fatty acid metabolism [133,139,140]. Interestingly, several PMP2 mutations were shown to cause a demyelinating form of Charcot–Marie–Tooth disease [141,142,143]; and upregulation of axonal neuregulin signaling causes an increase in PMP2 expression [144,145].

Circulating fatty acids are incorporated into adult myelin [146,147], and a few studies suggest that myelinating cells rely on fatty acid uptake for myelin biosynthesis [148,149,150]. However, because fatty acid uptake can be passive and because most fatty acids can be synthesized by oligodendrocytes and Schwann cells, the reliance of myelinating cells on fatty acid uptake is difficult to study. Essential fatty acids, linoleic acid and alpha-linolenic acid, cannot be synthesized by mammals. Excluding linoleic and alpha-linolenic acids from the diet of animals from their conception to 120 days of age altered the fatty acid composition of myelin and caused myelin splitting, but myelination remained relatively normal [151]. Similarly, excluding all fat from the diet of animals minimally alters the fatty acid composition of the CNS [152].

Recently, fatty acid uptake was proposed to compensate partially for deficiencies in fatty acid synthesis in both the CNS and the PNS [110,111]. Compensation by fatty acid uptake was more evident in myelinating cells in direct proximity to blood vessels or indirectly from an increase in horizontal flux of fatty acids through astrocytes or adipocytes [33,110]. Consequently, an increase or alteration in dietary fatty acids was proposed as a potential therapeutic strategy for diseases related to myelin formation. Notably, a few recent studies on mouse models for Pelizaeus–Merzbacher or Charcot–Marie–Tooth diseases have shown improvement in myelination when treated with custom lipid diet [153,154,155,156,157]. The mouse model for Pelizaeus–Merzbacher treated with a ketogenic diet for 10 weeks showed reduced axonal degeneration and normalization of motor functions. The mouse model for Charcot–Marie–Tooth disease 1A treated with an enriched phospholipid diet for 20 days during myelination or for 12 weeks after completion of myelin, leads to a marked amelioration of neuropathic symptoms. However, while high-fat diet can partially compensate for a deficiency in fatty acid synthesis in the CNS, in the PNS a high fat diet results in worsening of myelin defects when fatty acid synthesis is impaired [110,111]. The mechanisms underlying differential effects in the CNS vs. PNS remain to be determined. Taken together the current literature suggests that the uptake of fatty acid may not be the primary pathway for myelin formation. However, the importance of fatty acid uptake contributing to the energetic metabolism of myelinating cells and axonal trophic support is still unclear.

Finally, there is also new evidence that lipids could be provided to myelin cells by neurons. A recent study found that fatty acids are released by hyperactive neurons and can be taken up by neighboring glia to protect neurons from fatty acid toxicity [158].

## 11. Fatty Acid Oxidation

The nervous system has very high metabolic demands. Notably the CNS consumes 20% of the body’s oxygen supply. While the metabolic requirements for neural activity have been well characterized, the energetic requirement for myelin formation and maintenance, as well as the mechanisms for energetic production in myelinating cells, are not well understood [159]. Fatty acid oxidation is the mitochondrial aerobic process of breaking down a fatty acid into acetyl-CoA units (Figure 2). The total energy yield from oxidizing one molecule of fatty acid with 16 carbons leads to a total of 129 ATPs, over three times the amount of energy obtained from metabolizing a single molecule of glucose. However, fatty acid oxidation is slower, consumes more oxygen than glucose oxidation, and is a prominent source of reactive oxygen species generation.

Because myelinating cells need to produce high levels of fatty acids for generation of myelin lipids, there is a long-standing belief that oligodendrocytes and Schwann cells metabolism do not favor fatty acid oxidation [160] Some studies have reported that about 20% of the total energy expenses of the adult brain are spent during fatty acid oxidation, and it is generally believed that fatty acid oxidation occurs exclusively in astrocytes [161,162,163]. However, it was suggested that the energetic profile of myelinating cells is similar to the profile observed in astrocytes [109,164]. Thus, it is possible that in oligodendrocytes and Schwann cells both fatty acid oxidation and aerobic glycolysis exist simultaneously, and regulate each other [165]. No studies have investigated whether fatty acid oxidation is required to accommodate the energetic requirement of myelinating cells. Viader et al. reported that mitochondrial dysfunction in Schwann cells causes demyelination and axonal degeneration. This phenotype is presumably caused by (i) the toxic accumulation of acylcarnitine, an intermediate of fatty acid oxidation, and the disruption of the integrated stress response and, (ii) through an increase in fatty acid oxidation [115]. In contrast, Cermenati at al. reported that mice lacking SREBF1 exhibit a decrease in fatty acid synthesis and an increase in fatty oxidation. In spite of this, myelin defects in these animals are limited to an increase in myelin thickness and Remak bundles alterations [112].

## 12. Conclusions

Myelination is a highly demanding metabolic process, requiring oligodendrocytes and Schwann cells to precisely increase and coordinate RNA, protein and lipid synthesis, protein trafficking, to allow for their extensive membrane production. In addition to insulating axons, reports have begun to describe the emerging role of oligodendrocytes and Schwann cells in the metabolic support of axons [114,115,164,166,167,168,169,170]. In these instances, abnormal oligodendrocyte or Schwann cell metabolism leads to axon degeneration in addition to direct effects on myelinating cells. While we have now a broad understanding of the lipids utilized by myelinating cells, we still know very little about their building blocks, the fatty acids. The next challenge for the field will be to better understand how the fatty acid metabolism in oligodendrocytes and Schwann cells are regulated, how they regulate differentiation of myelinating cells, and how they contribute to the energetic and trophic support of axons. These principles may be translated into therapeutic opportunities for neuropathies either associated with myelin deficits or extended to disorders associated with white matter defects [171,172].

## Figures and Tables

**Table 1:** Comparison of the lipid composition of peripheral nervous system (PNS) and central nervous system (CNS) myelin. Myelin in the adult bovine spinal root and brain. ^a^ O’brien et al. 1967 [27]; ^b^ Norton and Poduslo 1973 [28].

	PNS^a^	CNS^b^
Cholesterol	41%	46%
Glycolipid	11%	20%
↳ Galactosylceramide	10%	17%
↳ Sulfatide	1%	3%
Phospholipid	29%	26%
↳ Plasmalogen	12%	13%
↳ Phosphatidylcholine	10%	7%
↳ Other Phospholipid	7%	7%
Sphingomyelin	13%	6%
Other lipids	6%	2%
